# Blood Flow Velocities as Determined by Real‐Time Phase‐Contrast MRI in Patients With Carotid Artery Stenosis

**DOI:** 10.1111/jon.70016

**Published:** 2025-02-24

**Authors:** Deborah Bochert, Sabine Hofer, Peter Dechent, Jens Frahm, Mathias Bähr, Jan Liman, Ilko Maier

**Affiliations:** ^1^ Department of Neurology University Medical Center Göttingen Gottingen Niedersachsen Germany; ^2^ Department of Cognitive Neurology University Medical Center Göttingen Gottingen Niedersachsen Germany; ^3^ Biomedical NMR Max Planck Institute for Multidisciplinary Sciences Gottingen Niedersachsen Germany; ^4^ Department of Neurology Paracelsus Medical School Nuremberg Bayern Germany

**Keywords:** atherosclerosis, blood flow, carotid stenosis, real‐time phase‐contrast MRI

## Abstract

**Background and Purpose:**

Real‐time phase‐contrast (RT‐PC) flow MRI can be used to determine quantitative flow parameters throughout the vessel lumen of extracranial, brain‐supplying arteries. Its potential value in the diagnostic workup of patients with carotid artery stenosis has not been evaluated.

**Methods:**

RT‐PC flow MRI was performed in 10 patients with carotid stenosis in comparison to conventional neurovascular ultrasound (nvUS). Peak systolic velocity, end‐diastolic velocity, mean flow velocity, and flow volumes have been evaluated by RT‐PC flow MRI. Measurements have been performed at standardized sites along the common, internal, and external carotid arteries on both sides and at the maximum of the carotid stenosis.

**Results:**

Blood flow velocities were significantly lower with RT‐PC flow MRI compared to nvUS and not consistently correlated between both methods. Within the maximum of the carotid stenosis, RT‐PC flow MRI showed implausible flow velocity reductions compared to nvUS. In contrast, the flow volumes determined by RT‐PC flow MRI—with exception of the stenosis maximum—were comparable with nvUS and significantly correlated in the prestenotic common carotid artery.

**Conclusion:**

RT‐PC flow MRI does not appear to be suitable for quantifying blood flow velocities and volumes in the patients with carotid stenosis compared to nvUS. Apart from the lower temporal resolution of RT‐PC MRI, the lack of correlation of blood flow velocities might be ascribed to the prevalence of nonlaminar flow within and behind the stenosis, which violates a general prerequisite for valid flow velocity measurements by PC MRI.

## 1 Introduction

Ischemic strokes are one of the leading causes of death and disability in adulthood worldwide [1]. Approximately 15% of ischemic strokes are caused by stenosis or occlusion of the carotid artery due to arterioarterial embolism or hemodynamic ischemia [2]. In order to prevent recurrent strokes, symptomatic carotid stenoses should be treated using carotid endarterectomy or endovascular intervention by carotid artery stenting in addition to best medical treatment [3].

Arteriosclerotic lesions of the carotid arteries can be assessed using neurovascular ultrasound (nvUS). This dynamic imaging modality is based on a frequency shift in the ultrasound beams reflected by blood cells, which correlates with their velocity (Doppler effect) and has a high temporal and special resolution. Thus, it is possible to examine the blood flow velocities as well as the morphology of the vessels and to assess the presence, degree, and localization of stenoses [4]. Due to its noninvasiveness and frequent availability, nvUS is the best‐established method in clinical practice for assessing carotid arteries. However, the assessment of spatial flow patterns in vessels is methodologically limited and furthermore depends on the experience of the examiner. Anatomical characteristics such as a short neck, obesity, high carotid bifurcation, or the skull bone can also affect the informative value of the nvUS, especially concerning the examination of the distal and intracranial parts of the internal carotid artery (ICA), which are typically not accessible by nvUS [5].

In addition to nvUS, magnetic resonance angiography (MRA) is one of the recommended complementary imaging methods in patients with ischemic stroke. Hereby, phase‐contrast (PC) MRA—in contrast to static time‐of‐flight‐(TOF)‐MRA—is a flow‐dependent examination technique that enables three‐dimensional visualization of blood flow [6]. PC‐MRA can be used to quantify velocities, volumes, and a detailed distribution of through‐plane velocities over the actual vessel lumen [7]. However, this technique requires multiple measurements, which significantly increase the measurement time [6]. Furthermore, it is based on electrocardiogram‐synchronized flow MRI acquisitions that combine data from multiple heartbeats and therefore bears the risk that relevant information could be lost by averaging incoherent MRI phases from nonperiodic heartbeats [8]. In particular, turbulent flows, which are indicative of bifurcation or stenosis, may not be adequately assessed and, as a result, stenoses may not be reliably visualized or underestimated.

Real‐time phase‐contrast (RT‐PC) MRI is an alternative method to potentially ameliorate the mentioned limitations. As previously described, the method employs a newer model‐based RT‐PC flow MRI reconstruction technique that yields velocity maps with excellent spatial and temporal accuracy, high velocity‐to‐noise ratio, and nearly noiseless floor of zero‐flow regions. It allows for a temporal resolution of 40 ms and in‐plane resolution of 0.8 mm for flow studies of the carotid artery—without electrocardiogram synchronization or restrictions on the patient's breathing [9, 10]. The technique is a PC flow adaptation of a real‐time MRI technique offering dynamic imaging at 20‐ms resolution and may be used for large cardiac vessels, peripheral veins, or cerebrospinal fluid [11, 12, 13, 14, 15, 16, 17]. In healthy volunteers, RT‐PC flow MRI of the carotid arteries is feasible, provides similar results to nvUS, and allows to assess the vessels supplying the brain with two‐dimensional velocity profiles [18]. Therefore, model‐based RT‐PC flow MRI could enable the detection of pathologic changes in cerebral vessels and further the evaluation of velocity profiles in the carotid arteries associated with various pathologies such as the hemodynamic effects of arteriosclerotic lesions including carotid stenosis of various degrees.

Aim of this study is to compare blood flow velocity and flow volume measurements performed by RT‐PC flow MRI to the reference method of nvUS and to qualitatively analyze intra‐, pre‐, and post‐stenosis three‐dimensional flow characteristics derived by this novel imaging technique.

## 2 Methods

### 2.1 Subjects

For this monocentric, prospective study, 10 subjects with unilateral carotid stenosis were recruited, being treated in the Department of Neurology of the University Medical Center Göttingen. Subjects <18 years of age, those who were unable to give informed consent, and individuals meeting general exclusion criteria for MRI were excluded from the study. Heart rate and blood pressure were measured before an nvUS was performed directly prior to the MRI examination within 2 h. The study was approved by the ethics committee of the University Medicine Göttingen (11/8/16), and all subjects gave written informed consent. This study was in consent with the Declaration of Helsinki.

### 2.2 RT‐PC Flow MRI

MRI examinations were conducted at 3T (Prisma Fit; Siemens Healthineers, Erlangen, Germany) using a 64‐channel head‐neck array coil. RT‐PC flow MRI was performed as previously described [16, 17] based on a highly undersampled radial FLASH sequence with asymmetric echoes and sequential flow encoding in combination with a model‐based reconstruction technique [9, 10]. Identical methodology had been applied as in a previous study on RT‐PC MRA measurements of the carotid arteries in healthy subjects [18], including online reconstruction and display of real‐time velocity maps, model‐based reconstruction offline after real‐time data acquisition on a single graphics processing unit (GeForce GTX 580; NVIDIA, Santa Clara, CA), and through‐plane flow measurements to achieve a total acquisition time of 40 ms for a pair of images with and without flow‐encoding gradients.

**FIGURE 1:**
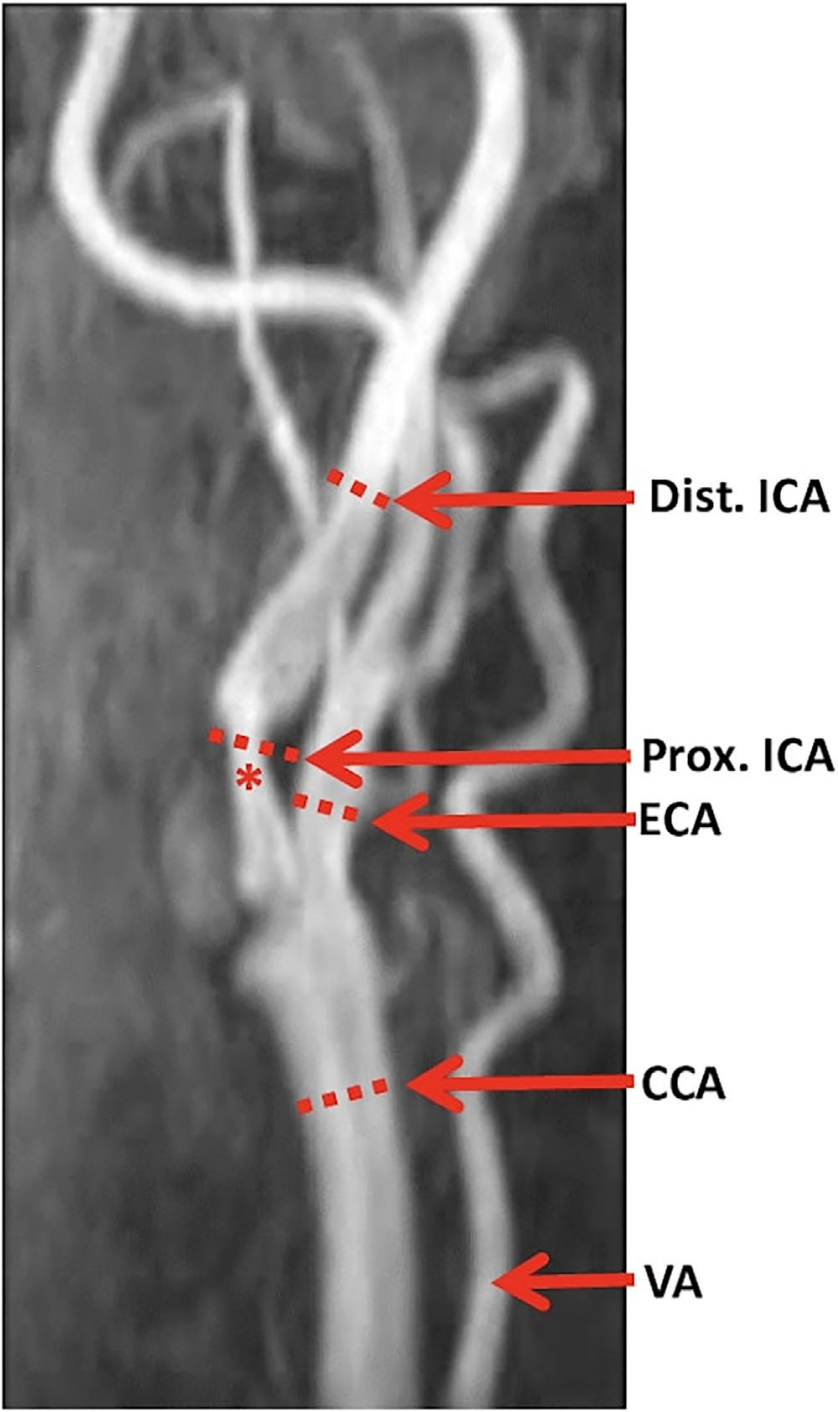
Sagittal time‐of‐flight MR angiogram indicating locations for flow measurements in the common carotid artery (CCA), internal carotid artery (ICA), and external carotid artery (ECA): CCA 12 mm proximal to the carotid sinus, proximal ICA and ECA 12 mm distal to the carotid bifurcation (in case of site of ICA stenosis measurement has been performed at the site of the stenosis maximum), and distal ICA at the angle of the jaw. Note the measurement site of the maximum of the stenosis in the proximal ICA (*). VA, vertebral artery; Dist., distal; Prox., proximal.

RT‐PC flow MRI data were measured during free breathing for a period of 15 s. Conventional TOF‐MRA was used to depict the common carotid artery (CCA), ICA, and external carotid artery (ECA). Flow measurements were conducted at four standardized locations: CCA 12 mm below the carotid sinus, proximal ICA and ECA 12 mm above the carotid bifurcation, and distal ICA above the proximal ICA. TOF‐MRA was also used to determine the maximum of the carotid stenosis, at where the measurements for the flow velocities in the carotid stenosis were performed (see example in Figure 1).

Quantitative analyses of magnitude images and velocity maps were achieved using a software prototype developed specifically for the processing of real‐time MRI data (Fraunhofer MEVIS, Bremen, Germany). This enables fully automated vessel segmentation and flow analysis of RT‐PC flow MRA data throughout the entire time series. An accurate assessment of the vessel lumen is achieved by the improved spatial and temporal accuracy of the serial magnitude images and velocity maps as well as the dynamic propagation of the calculated deformation fields [19]. Partial volume effects are therefore minimized. Flow parameters obtained include peak velocities, velocities averaged over the vessel lumen, vessel areas, and flow volumes. Three‐dimensional representations of the velocity profiles were generated using MATLAB (v. 9.13.0; MathWorks, Natick, MA, USA, https://www.mathworks.com).

### 2.3 nvUS of Carotid Arteries

Ultrasound of the carotid arteries was examined with a GE Logique s8 system using brightness‐mode imaging, pulsed duplex scanning, and color Doppler flow imaging at 8–12 MHz and was performed by a specialist with more than 5 years of experience who is certified by Deutsche Gesellschaft für Ultraschall in der Medizin. To verify the reliability of the velocity measurements, a second ultrasound examination was repeated by specialist with more than 10 years of nvUS experience. The standardized nvUS protocol was performed as previously described [18].

### 2.4 Statistical Analysis

The statistical analysis was performed with SPSS 21 (IBM SPSS Statistics, Armonk, NY, USA, https://www.ibm.com). Baseline data for all subjects are expressed as mean ± standard deviation (SD). Peak systolic velocity (PSV), end‐diastolic velocity (EDV), mean flow velocity (MFV), and flow volume were calculated for each site on the left and right side. Comparison of mean flow velocities, flow volumes, and vessel areas (vertical plane) of both sides was conducted using a *t*‐test for dependent samples. Correlations between the flow velocities measured by RT‐PC flow MRI and nvUS were evaluated by a bivariate Pearson correlation. The *p*‐values below 0.05 were considered statistically significant.

## 3 Results

The baseline characteristics of the 10 subjects are shown in Table 1. The majority of the subjects were male, and the mean age was 69.5 ± 8.5 years. Blood pressure and heart rate before nvUS were within normal ranges. All subjects were reported to have arterial hypertension. Four patients had hyperlipidemia, and one patient each had documented diabetes mellitus or nicotine consumption.

**TABLE 1:** Baseline characteristics.

Parameter	
Age (years)	69.5 ± 8.5
Sex (male, %)	7 (70)
Heart rate (min^−1^)	71.1 ± 15.5
Systolic blood pressure (mmHg)	136.6 ± 11.3
Diastolic blood pressure (mmHg)	73.9 ± 9.2
Weight (kg)	80.5 ± 9.8
Height (cm)	169.1 ± 9.0
Arterial hypertension (*n*, %)	10 (100)
Diabetes mellitus (*n*, %)	1 (10)
Hyperlipidemia (*n*, %)	4 (40)
Ipsilateral carotid stenosis degree NASCET (%)	50 (50, 50)
Ipsilateral carotid stenosis degree ECST (%)	70 (70, 70)
Symptomatic carotid stenosis (*n*, %)	3 (30)

*Note*: Characteristics of studied patients (*n* = 10). Values are described as mean ± standard deviation unless otherwise indicated, except for ipsilateral carotid stenosis degree, which is given as median (interquartile range) and was determined by duplex sonography.

Abbreviations: ECST, European Carotid Surgery Trial; *n*, quantity of subjects; NASCET, North American Symptomatic Carotid Endarterectomy Trial.

### 3.1 Qualitative Comparison of RT‐PC Flow MRI and nvUS

**FIGURE 2:**
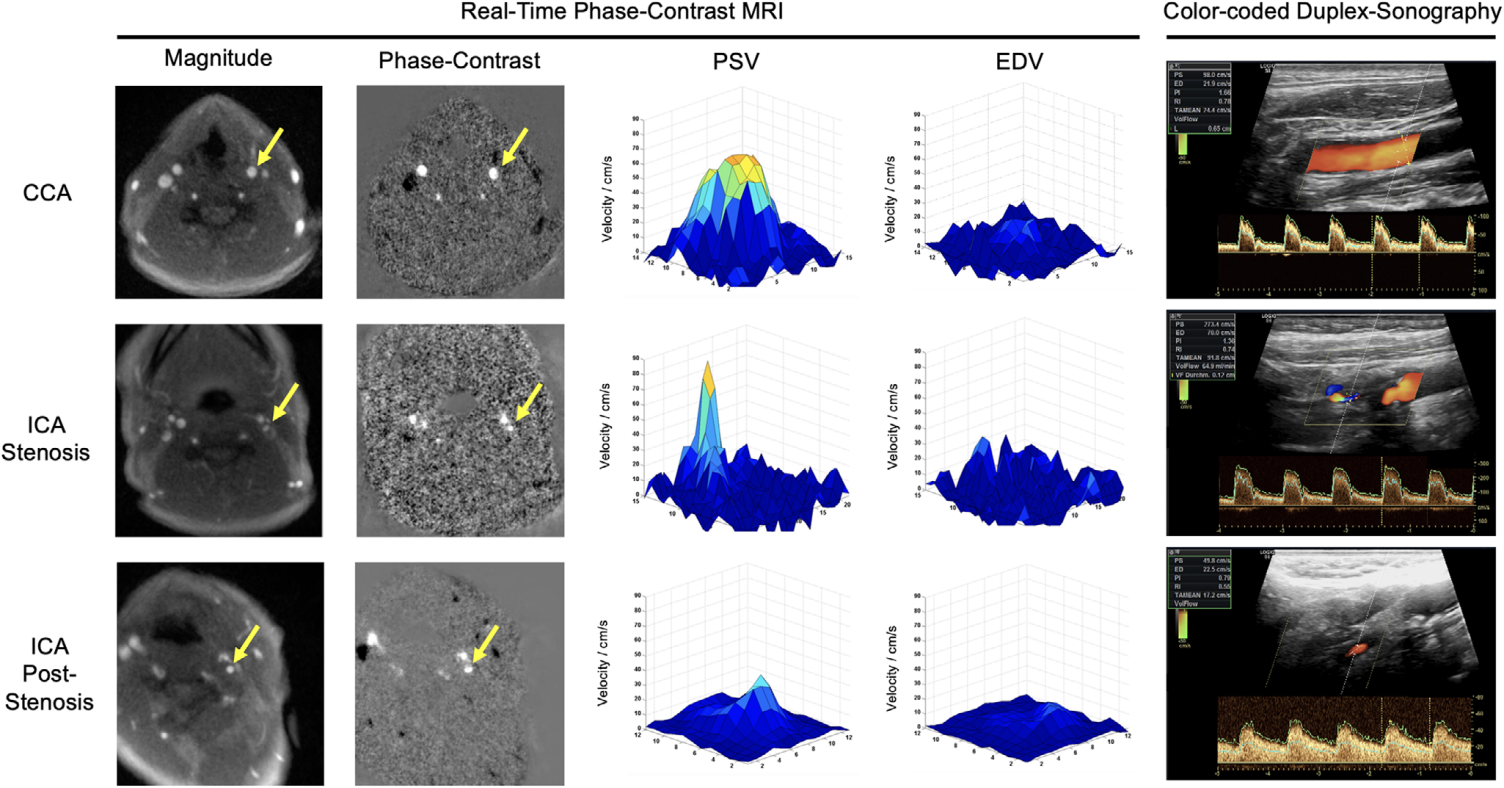
Patient with high‐grade carotid artery stenosis: Representative transversal real‐time phase‐contrast MRI images of the anatomy (magnitude images) and velocity‐coded phase‐contrast images, three‐dimensional representations of the velocities in plane at peak systole (PSV) and end diastole (EDV), as well as representative longitudinal color‐coded duplex sonography images at the common carotid artery (CCA) and internal carotid artery (ICA) at the maximum of the stenosis and post‐stenosis. In the magnitude images, all arteries and veins are hyperintense; in the phase‐contrast images, arteries are hyperintense (upward flow) and veins are hypointense (downward flow). The yellow arrows mark the left CCA, intra‐stenotic ICA, and post‐stenotic ICA. Note the increased pulsatility in the CCA (pre‐stenotic flow pattern) and decreased pulsatility of the distal ICA (post‐stenotic flow pattern). PS, peak systole; ED, end diastole; PI, pulsatility index; RI, resistance index; TAMEAN, time‐averaged mean velocity; Durchm, diameter.

**FIGURE 3:**
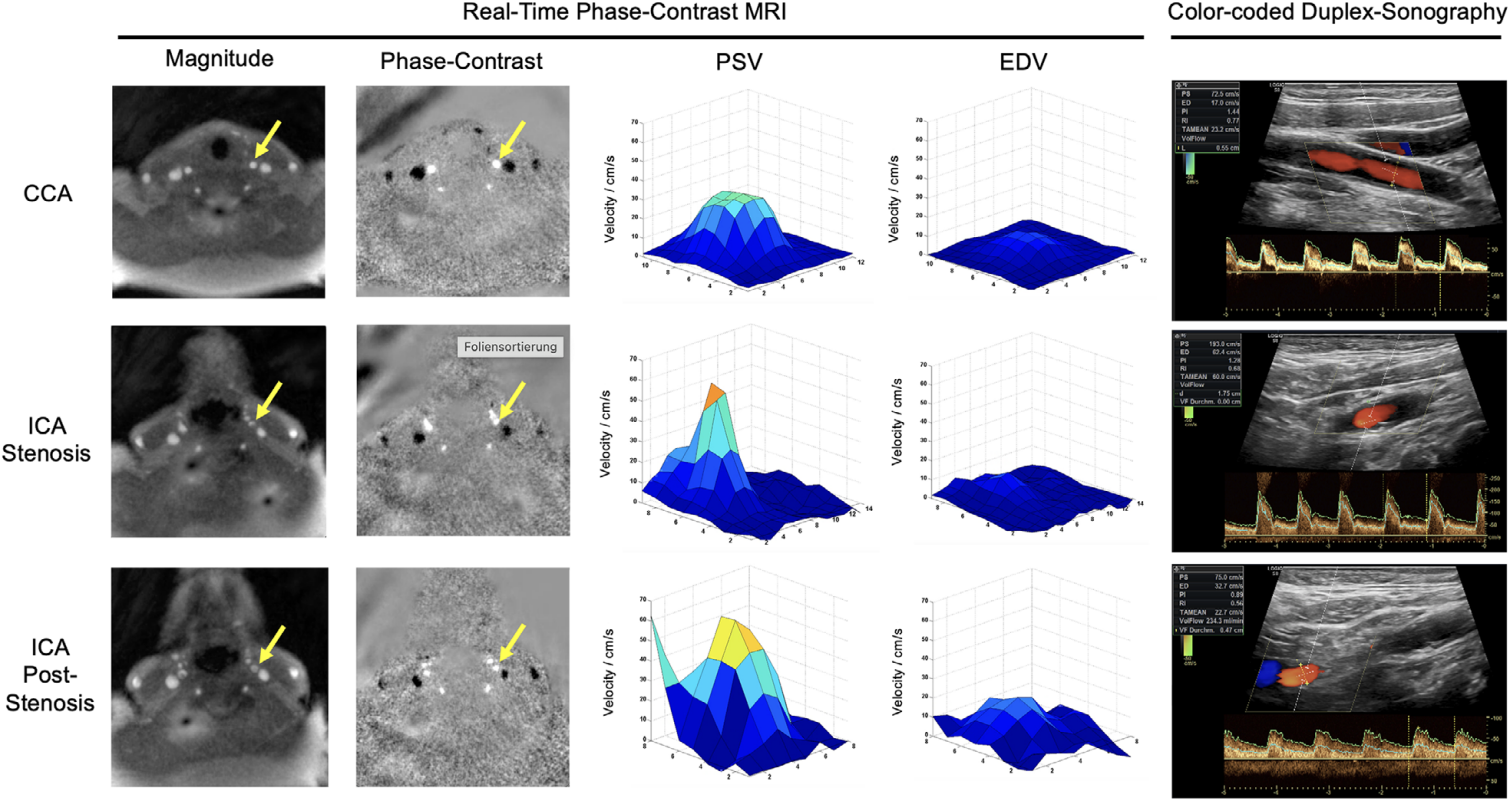
Patient with low‐grade carotid artery stenosis: Representative transversal real‐time phase‐contrast MRI images of the anatomy (magnitude images) and velocity‐coded phase‐contrast images, three‐dimensional representations of the velocities in plane at peak systole (PSV) and end diastole (EDV), as well as representative longitudinal color‐coded duplex sonography images at the common carotid artery (CCA) and internal carotid artery (ICA) at the maximum of the stenosis and post‐stenosis. In the magnitude images, all arteries and veins are hyperintense; in the phase‐contrast images, arteries are hyperintense (upward flow) and veins are hypointense (downward flow). The yellow arrows mark the left CCA, intra‐stenotic ICA, and post‐stenotic ICA. Note the lower pulsatility in the CCA and the higher PSV in the post‐stenotic ICA compared to the patient shown in Figure 2 with a higher grade ICA stenosis. PS, peak systole; ED, end diastole; PI, pulsatility index; RI, resistance index; TAMEAN, time‐averaged mean velocity; Durchm, diameter.

Representative RT‐PC MRI images of the anatomy (magnitude images), velocities (PC maps), and three‐dimensional representations of the systolic and diastolic velocity distributions of the ICA and CCA are shown in Figure 2 (high‐grade stenosis) and Figure 3 (low‐grade stenosis). In these examples, the CCA shows a physiological, centrally flattened velocity profile during systole and diastole. In contrast, the ICA showed a steep increase in systolic flow velocity within the stenosis, which is more distinct in the patient with high‐grade ICA stenosis (Figure 2). In both representative examples, the significant reduction of the perfused lumen in the area of the stenosis is evident. After the stenosis, PSV was significantly reduced in the patient with high‐grade carotid stenosis (Figure 2), while PSV was higher in the patient with medium‐grade carotid stenosis (Figure 3).

RT‐PC flow MRI movies of the anatomy (magnitude images) and velocities (PC maps) as well as three‐dimensional representations within the carotid stenosis of the same cases as in Figures 2 and 3 can be found as videos (Videos 1A, 1B, 1C, 2A, 2B, 2C).

**VIDEO 1A:** Representative transversal real‐time phase‐contrast MRI images of the anatomy (magnitude images) of the patient shown in Figure 2. Video content can be viewed at https://onlinelibrary.wiley.com/doi/10.1111/jon.70016.

**VIDEO 1B:** Representative transversal real‐time phase‐contrast MRI images of the patient shown in Figure 2. Video content can be viewed at https://onlinelibrary.wiley.com/doi/10.1111/jon.70016.

**VIDEO 1C:** Representative velocity‐coded phase‐contrast images and three‐dimensional representations of the velocities in plane of the patient shown in Figure 2. Video content can be viewed at https://onlinelibrary.wiley.com/doi/10.1111/jon.70016.

**VIDEO 2A:** Representative transversal real‐time phase‐contrast MRI images of the anatomy (magnitude images) of the patient shown in Figure 3. Video content can be viewed at https://onlinelibrary.wiley.com/doi/10.1111/jon.70016.

**VIDEO 2B:** Representative transversal real‐time phase‐contrast MRI images of the patient shown in Figure 3. Video content can be viewed at https://onlinelibrary.wiley.com/doi/10.1111/jon.70016.

**VIDEO 2C:** Representative velocity‐coded phase‐contrast images and three‐dimensional representations of the velocities in plane of the patient shown in Figure 3. Video content can be viewed at https://onlinelibrary.wiley.com/doi/10.1111/jon.70016.

### 3.2 Quantitative Comparison of RT‐PC Flow MRI and nvUS

Table 2 shows the quantitative differences between PSV, EDV, and flow volumes in the CCA and ICA between RT‐PC MRI and nvUS. PSV, EDV, and MFV all were significantly lower using RT‐PC MRI compared to nvUS. In particular, the highest difference between blood flow velocities determined by RT‐PC MRI and nvUS has been found in the maximum of the stenosis (mean difference 146.6 ± 43.5, *p* < 0.001). Averaged over all locations, the MRI‐derived values for PSV, EDV, and MFV (48, 11.4, and 15.2 cm/s, respectively) were 47.5%, 39%, and 51.2% lower compared to the velocities determined by nvUS.

**TABLE 2:** Mean velocities and flow volumes using real‐time phase‐contrast MRI and neurovascular ultrasound.

Vessel localization	RT‐PC MRI (mean cm/s ± SD)	nvUS (mean cm/s ± SD)	*p*‐value^a^	Difference (cm/s ± SD, %)	95% CI
**PSV (cm/s)**					
CCA ipsilateral to stenosis	45.6 ± 12.5	68.3 ± 14.9	0.001	22.8 ± 15.7 (33.4)	11.6, 34.0
CCA contralateral to stenosis	56.5 ± 11.3	79.5 ± 20.8	0.005	23.0 ± 19.6 (28.9)	9.0, 37.0
ICA stenosis maximum	75.5 ± 31.7	222.2 ± 41.7	<0.001	146.6 ± 43.5 (66.0)	115.5, 177.7
ICA contralateral to stenosis	45.7 ± 9.1	79.4 ± 25.1	<0.001	33.6 ± 21.3 (42.3)	18.4, 48.9
ICA distal/post‐stenosis	46.9 ± 11.6	78.9 ± 30.1	0.017	32.0 ± 34.7 (40.6)	7.1, 56.8
ICA distal contralateral	48.3 ± 5.5	78.2 ± 20.6	0.001	29.9 ± 20.9 (38.2)	15.0, 44.9
Mean difference overall (cm/s, %)				48.0 (47.5)	
**EDV (cm/s)**					
CCA ipsilateral to stenosis	13.8 ± 4.4	16.6 ± 5.6	0.090	2.8 ± 4.6 (16.9)	−0.5, 6.1
CCA contralateral to stenosis	16.5 ± 3.2	20.0 ± 5.5	0.042	3.5 ± 4.6 (17.5)	0.2, 6.8
ICA stenosis maximum	27.5 ± 9.7	65.1 ± 28.0	0.005	37.6 ± 32.7 (57.8)	14.2, 61.0
ICA contralateral to stenosis	15.4 ± 2.0	23.6 ± 5.9	0.003	8.2 ± 6.5 (34.7)	3.5, 12.8
ICA distal/post‐stenosis	16.1 ± 4.1	23.9 ± 7.3	0.004	7.9 ± 6.5 (33.1)	3.2, 12.5
ICA distal contralateral	17.9 ± 2.5	26.5 ± 6.3	0.010	8.6 ± 8.3 (32.5)	2.6, 14.5
Mean difference overall (cm/s, %)				11.4 (39.0)	
**Flow volume (ml/min)**					
CCA ipsilateral to stenosis	312.0 ± 105.6	306.4 ± 140.2	0.829	−5.6 ± 79.7 (−1.8)	−62.6, 51.4
CCA contralateral to stenosis	372.0 ± 99.3	363.3 ± 124.3	0.746	−8.7 ± 82.0 (−2.4)	−67.4, 50.0
ICA stenosis maximum	186.0 ± 50.6	289.6 ± 127.4	0.022	103.6 ± 118.4 (35.8)	18.9, 188.3
ICA contralateral to stenosis	209.0 ± 42.3	208.8 ± 86.4	0.993	−0.2 ± 89.7 (−0.1)	−64.4, 63.9
ICA distal/post‐stenosis	184.0 ± 49.5	167.2 ± 95.5	0.561	−16.8 ± 88.3 (−10.0)	−80.0, 46.3
ICA distal contralateral	219.0 ± 40.9	185.5 ± 60.5	0.244	−33.5 ± 85.0 (−18.0)	−94.3, 27.3
Mean difference overall (mL/min, %)				6.5 (2.6)	

*Note*: Values are averaged across subjects and given as mean ± standard deviation (SD)

Abbreviations: CCA, common carotid artery; CI, confidence interval; EDV, end‐diastolic velocity; ICA, internal carotid artery; MRI, magnetic resonance imaging; nvUS, neurovascular ultrasound; PSV, peak systolic velocity; RT‐PC, real‐time phase‐contrast.

^a^ *t*‐test.

The flow volumes measured in the carotid stenosis also were significantly lower using RT‐PC MRI (mean difference 103.6 ± 118.4), while all other flow volume measurements were comparable between both methods and being consistently statistically nonsignificantly lower compared to nvUS.

### 3.3 Correlations of Blood Flow Velocities and Flow Volumes Between RT‐PC Flow MRI and nvUS

There were no consistent positive correlation coefficients for most of the blood flow velocity measurements derived by RT‐PC MRI and nvUS (Table 3). All flow velocities except ICA‐PSV contralateral to the stenosis (*r* = 0.685, *p* = 0.029) and CCA‐MFV ipsilateral to stenosis (*r* = 0.810, *p* = 0.005) showed no significant positive correlation between the two methods. Flow volumes were only correlated in the CCA both ipsilaterally (*r* = 0.826, *p* = 0.003) and contralaterally (*r* = 0.685, *p* = 0.029) to the ICA stenosis.

**TABLE 3:** Correlations between real‐time phase‐contrast MRI and neurovascular ultrasound.

Vessel localization	Correlation coefficient	*p*‐value^a^
**PSV (cm/s)**		
CCA ipsilateral to stenosis	0.357	0.312
CCA contralateral to stenosis	0.394	0.260
ICA stenosis maximum	0.324	0.361
ICA contralateral to stenosis	0.685	0.029
ICA distal/post‐stenosis	−0.239	0.507
ICA distal contralateral	0.127	0.726
**EDV (cm/s)**		
CCA ipsilateral to stenosis	0.589	0.073
CCA contralateral to stenosis	0.353	0.318
ICA stenosis maximum	−0.349	0.323
ICA contralateral to stenosis	0.067	0.855
ICA distal/post‐stenosis	0.452	0.190
ICA distal contralateral	−0.515	0.128
**Flow volume (ml/min)**		
CCA ipsilateral to stenosis	0.826	0.003
CCA contralateral to stenosis	0.685	0.029
ICA stenosis maximum	0.370	0.293
ICA contralateral to stenosis	0.512	0.130
ICA distal/post‐stenosis	0.400	0.252
ICA distal contralateral	−0.366	0.298

*Note*: Values are averaged across subjects.

Abbreviations: CCA, common carotid artery; EDV, end‐diastolic velocity; ICA, internal carotid artery; nvUS, neurovascular ultrasound; PSV, peak systolic velocity.

^a^ Spearman correlation.

## 4 Discussion

RT‐PC flow MRI is a promising method to quantify blood flow velocities in the brain‐supplying arteries, especially in areas where other techniques like nvUS are not applicable. In this study, we could not demonstrate comparable measurements of blood flow velocities between RT‐PC MRI and nvUS in patients with carotid stenosis. Overall, blood flow velocities determined by RT‐PC MRI were significantly lower compared to nvUS, and, moreover, there were no positive correlations between the measurements. These findings could be explained by multiple methodological and physical differences between these two imaging techniques.

### 4.1 Laminar Versus Nonlaminar Flow

In a previous study, RT‐PC flow MRI was evaluated as a new imaging technique against the reference method of nvUS in healthy volunteers without stenosis of the carotid arteries. This study also found lower blood flow velocities determined by the RT‐PC MRI compared to nvUS but was able to demonstrate positive correlations between the two techniques. In addition, as in the present study, it was possible to show detailed characterization of the brain‐supplying arteries by dynamic velocity profiles throughout the entire vessel lumen and characterize blood flow abnormalities and flow disturbances in the Doppler spectrum as well as in the three‐dimensional representation of the through‐plane flow in the corresponding imaging localizations [18].

### 4.2 Temporal resolution

In the present study, we confirmed a significant lower blood flow velocity measurements using RT‐PC MRI compared to nvUS [18]. As in nvUS, the PSV can be easily determined as the maximum of the Doppler spectrum in the systole; however, the lower temporal resolution of RT‐PC flow MRI of 40 ms/image might miss the peak velocity during systole and average the high velocities toward lower values. This particularly holds true when investigating flow spectra with a very short maximum PSV, which can be found in vessels with higher peripheral resistance like in the CCA or ECA. In comparison to RT‐PC MRI, the temporal resolution of nvUS is much higher given the propagation of sound waves in soft tissue with around 1540 m/s and the insonation depth of the carotid arteries of just a few centimeters [20].

### 4.3 Partial Volume Effect

Through‐plane flow has been measured with RT‐PC MRI with a 90° angle to the corresponding artery. In contrast, in nvUS, blood flow velocities are measured using a Doppler volume being placed longitudinally and via angle correction into the corresponding vessel volume. Thus, in nvUS, partial volume effects or the measurement of the movement of the vessel walls or surrounding moving tissue are highly unlikely. On the other hand, when defining the vessel lumen to measure through‐plane flow in RT‐PC MRI, it is not entirely possible to determine where the vessel lumen ends and the vessel wall begins. Therefore, partial volume effects with the measurement of slow‐moving structures around the blood flow within the vessel lumen can lead to falsely lower blood flow velocities.

### 4.4 Physical Aspects

Furthermore, blood flow velocities measured by RT‐PC flow MRI and nvUS are sensitive to major physical differences between both techniques. nvUS measures the frequency shifts of ultrasound beams that are reflected by corporal parts of the blood flow and therefore detects the fast‐moving blood cells [21]. Moreover, blood flow velocity in nvUS is typically measured with angle correction at the center of the flowed‐through lumen and thus largely detects laminar flow (i.e., one constant velocity) during a very short period of time. MRI, on the other hand, measures the phase shifts of water proton spins moving through a magnetic field gradient, so that the slower plasma current is also acquired in addition to the corpuscular blood components. In the present study, PSV and EDV were also significantly lower in the proximal and distal ICA when determined by RT‐PC flow MRI. However, compared to the previous study in healthy volunteers [18], there was no significant correlation between nvUS and RT‐PC flow MRI regarding blood flow velocities. This may be related to nonlaminar blood flow profiles, as carotid stenosis certainly induces turbulence and flow separations [22, 23]. In preceding studies, discrepant cerebral blood flow (CBF) measurements between PC‐MRI and nvUS were attributed to methodological limitations in assessing “complex” blood flow patterns in large arteries [24, 25]. In fact, in PC flow MRI measurements, turbulent flow leads to intravoxel phase dispersion, partial phase cancellation, and corresponding signal void. Thus, for physical reasons, the validity of PC flow MRI is violated for nonlaminar flow.

It has been theorized that differences in vessel anatomy might have a significant impact on the measurement of CBF since studies on healthy subjects have shown that variability of CBF is higher between PC‐MRI and nvUS in the ICA than in the vertebral artery. Although blood flow velocity and vessel diameter in the vertebral artery significantly correlated between PC‐MRI and nvUS, no corresponding correlation was found in the ICA. The measured vessel diameter was significantly larger by approximately 12% using PC MRI. However, no differences in volumetric blood flow were observed between the methods in healthy subjects [26]. In the present study, flow volume in the CCA correlated between RT‐PC flow MRI and nvUS, but not in the area of ICA stenosis and post‐stenotic ICA. In healthy volunteers, there was no significant correlation in flow volume between the two methods, but the flow volume determined with RT‐PC flow MRI was higher [18]. This could be attributed to an accurate, user‐independent assessment of the vessel lumen. However, the accuracy seems to be limited in patients with carotid stenosis, as the flow volume within the carotid stenosis was significantly higher with nvUS than with RT‐PC MRI in the current study.

Previous studies have shown that RT‐PC flow MRI of the carotid arteries is applicable for the quantitative assessment of blood flow providing consistent blood flow velocities and volumes in healthy subjects. However, in the current study in patients with carotid stenosis, such behavior was not observable. The main reason for this finding is likely to be the prevalence of turbulent blood flow caused by the stenosis. This is because turbulent flow cannot be properly quantified by PC MRI as the method requires laminar flow to ensure constant velocities within an image voxel during the acquisition of a PC map. Therefore, the present study casts general doubt on the usefulness and clinical application of PC MRI in patients with stenosis of the brain‐supplying arteries. Nevertheless, RT‐PC flow MRI might be a valuable tool to (dynamically) assess flow volumes of brain‐supplying arteries without significant stenosis, especially intracranially, where the informative value of nvUS is often limited. Besides the possible diagnostic value of three‐dimensional, qualitative assessments of these nonstenotic brain‐supplying vessels, the problem of lower flow velocities derived by RT‐PC MRI may be addressed by assigning a nonlinear scaling factor taking lumen size and voxel size into consideration, as the latter seems to be a main factor behind these lower velocities.

In addition, major advantages of the RT‐PC MRI method include the absent need for external triggers and therefore the possibility to study patients with cardiac arrhythmias (such as atrial fibrillation). Future work should investigate intracranial blood flow in patients with and without occlusions of extracranial brain‐supplying arteries in an attempt to determine the hemodynamic relevance of the stenoses and the cerebral reserve capacity.

## Conflicts of Interest

JF is a co‐inventor of the real‐time MRI patent used here. The other authors declare no conflicts of interest.

## References

[1] C. J. L. Murray , T. Vos , R. Lozano , et al., “Disability‐Adjusted Life Years (DALYs) for 291 Diseases and Injuries in 21 Regions, 1990–2010: A Systematic Analysis for the Global Burden of Disease Study 2010,” The Lancet 380 (2012): 2197–2223. 10.1016/S0140-6736(12)61689-4 23245608

[2] F. Palm , C. Urbanek , J. Wolf , et al., “Etiology, Risk Factors and Sex Differences in Ischemic Stroke in the Ludwigshafen Stroke study, a Population‐Based Stroke Registry,” Cerebrovascular Diseases 33 (2012): 69–75. 22133999 10.1159/000333417

[3] D. O. Kleindorfer , A. Towfighi , S. Chaturvedi , et al., “2021 Guideline for the Prevention of Stroke in Patients with Stroke and Transient Ischemic Attack: A Guideline from the American Heart Association/American Stroke Association,” Stroke 52, no. 7 (2021): e364–e467. 34024117 10.1161/STR.0000000000000375

[4] E. I. Bluth , A. T. Stavros , K. W. Marich , S. M. Wetzner , D. Aufrichtig , and J. D. Baker , “Carotid Duplex Sonography: A Multicenter Recommendation for Standardized Imaging and Doppler Criteria,” Radiographics 8 (1988): 487–506. 3289100 10.1148/radiographics.8.3.3289100

[5] A. Herment , J. P. Guglielmi , P. Dumee , P. Peronneau , and P. Delouche , “Limitations of Ultrasound Imaging and Image Restoration,” Ultrasonics 25 (1987): 267–273. 3310352 10.1016/0041-624x(87)90048-5

[6] C. Roth , “Cerebrovascular Diagnostics—Imaging,” Der Radiologe 52 (2012): 1101–1106. 23178785 10.1007/s00117-012-2370-8

[7] Y. Tao , G. Rilling , M. Davies , and I. Marshall , “Carotid Blood Flow Measurement Accelerated by Compressed Sensing: Validation in Healthy Volunteers,” Magnetic Resonance Imaging 31 (2013): 1485–1491. 23830111 10.1016/j.mri.2013.05.009

[8] B. D. Gelfand , F. H. Epstein , and B. R. Blackman , “Spatial and Spectral Heterogeneity of Time‐Varying Shear Stress Profiles in the Carotid Bifurcation by Phase‐Contrast MRI,” Journal of Magnetic Resonance Imaging 24 (2006): 1386–1392. 17083089 10.1002/jmri.20765

[9] Z. Tan , V. Roeloffs , D. Voit , et al., “Model‐Based Reconstruction for Real‐Time Phase‐Contrast Flow MRI: Improved Spatiotemporal Accuracy,” Magnetic Resonance in Medicine 77 (2017): 1082–1093. 26949221 10.1002/mrm.26192

[10] Z. Tan , T. Hohage , O. Kalentev , et al., “An Eigenvalue Approach for the Automatic Scaling of Unknowns in Model‐Based Reconstructions: Application to Real‐Time Phase‐Contrast Flow MRI,” NMR in Biomedicine 30, no. 12 (2017): e3835. 10.1002/nbm.3835 28960554

[11] A. A. Joseph , K. D. Merboldt , D. Voit , J. Dahm , and J. Frahm , “Real‐Time Magnetic Resonance Imaging of Deep Venous Flow During Muscular Exercise‐Preliminary Experience,” Cardiovascular Diagnosis and Therapy 6 (2016): 473–481. 28123969 10.21037/cdt.2016.11.02 PMC5220202

[12] S. Dreha‐Kulaczewski , A. A. Joseph , K. D. Merboldt , H. C. Ludwig , J. Gärtner , and J. Frahm , “Identification of the Upward Movement of Human CSF in Vivo and Its Relation to the Brain Venous System,” Journal of Neuroscience 37 (2017): 2395–2402. 28137972 10.1523/JNEUROSCI.2754-16.2017 PMC6596847

[13] J. T. Kowallick , A. A. Joseph , C. Unterberg‐Buchwald , et al., “Real‐Time Phase‐Contrast Flow MRI of the Ascending Aorta and Superior Vena Cava as a Function of Intrathoracic Pressure (Valsalva Manoeuvre),” British Journal of Radiology 87 (2014): 20140401. 25074791 10.1259/bjr.20140401 PMC4170873

[14] M. Uecker , S. Zhang , D. Voit , A. Karaus , K. D. Merboldt , and J. Frahm , “Real‐Time MRI at a Resolution of 20 ms,” NMR in Biomedicine 23 (2010): 986–994. 20799371 10.1002/nbm.1585

[15] A. A. Joseph , J. T. Kowallick , K. D. Merboldt , et al., “Real‐Time Flow MRI of the Aorta at a Resolution of 40 msec,” Journal of Magnetic Resonance Imaging 40 (2014): 206–213. 24123295 10.1002/jmri.24328

[16] A. A. Joseph , K. D. Merboldt , D. Voit , et al., “Real‐Time Phase‐Contrast MRI of Cardiovascular Blood Flow Using Undersampled Radial Fast Low‐Angle Shot and Nonlinear Inverse Reconstruction,” NMR in Biomedicine 25 (2012): 917–924. 22180216 10.1002/nbm.1812

[17] M. Untenberger , Z. Tan , D. Voit , et al., “Advances in Real‐Time Phase‐Contrast Flow MRI Using Asymmetric Radial Gradient Echoes,” Magnetic Resonance in Medicine 75 (2016): 1901–1908. 26096085 10.1002/mrm.25696

[18] I. L. Maier , S. Hofer , A. A. Joseph , et al., “Carotid Artery Flow as Determined by Real‐Time Phase‐Contrast Flow MRI and Neurovascular Ultrasound: A Comparative Study of Healthy Subjects,” European Journal of Radiology 106 (2018): 38–45. 30150049 10.1016/j.ejrad.2018.07.011

[19] Chitiboi T , Hennemuth A , Tautz L , et al., “Context‐Based Segmentation and Analysis of Multi‐Cycle Real‐Time Cardiac MRI,” in *2014 IEEE 11th International Symposium on Biomedical Imaging, ISBI 2014* (New York: IEEE, 2014), 943–946.

[20] S. K. Edelman , “Propagation Speed and Distance Measurement,” Echocardiography 5 (1988): 71–77.

[21] R. E. Zierler , “Carotid Duplex Criteria: What Have We Learned in 40 Years?,” Seminars in Vascular Surgery 33 (2020): 36–46. 33308594 10.1053/j.semvascsurg.2020.05.003

[22] K. Jain , “The Effect of Varying Degrees of Stenosis on Transition to Turbulence in Oscillatory Flows,” Biomechanics and Modeling in Mechanobiology 21 (2022): 1029–1041. 35445319 10.1007/s10237-022-01579-0 PMC9132830

[23] M. Ziegler , J. Lantz , T. Ebbers , and P. Dyverfeldt , “Assessment of Turbulent Flow Effects on the Vessel Wall Using Four‐Dimensional Flow MRI,” Magnetic Resonance in Medicine 77 (2017): 2310–2319. 27350049 10.1002/mrm.26308

[24] S. Meckel , L. Leitner , L. H. Bonati , et al., “Intracranial Artery Velocity Measurement Using 4D PC MRI at 3 T: Comparison With Transcranial Ultrasound Techniques and 2D PC MRI,” Neuroradiology 55 (2013): 389–398. 23143179 10.1007/s00234-012-1103-z

[25] S. L. Peng , P. Su , F. N. Wang , et al., “Optimization of Phase‐Contrast MRI for the Quantification of Whole‐Brain Cerebral Blood Flow,” Journal of Magnetic Resonance Imaging 42 (2015): 1126–1133. 25676350 10.1002/jmri.24866 PMC4532651

[26] M. A. Khan , J. Liu , T. Tarumi , et al., “Measurement of Cerebral Blood Flow Using Phase Contrast Magnetic Resonance Imaging and Duplex Ultrasonography,” Journal of Cerebral Blood Flow and Metabolism 37 (2017): 541–549. 26873888 10.1177/0271678X16631149 PMC5381449

